# Perceived COVID-19 susceptibility and preventive behaviors: moderating effects of social support in Italy and South Korea

**DOI:** 10.1186/s12889-022-14866-3

**Published:** 2023-01-03

**Authors:** Soontae An, Peter J. Schulz, Hannah Kang

**Affiliations:** 1 Division of Communication and Media, Ewha Womans University, Seoul, 03760 South Korea; 2 Faculty of Communication, Culture and Society, Università della Svizzera italiana, Lugano, Switzerland; 3 Department of Politics and Communication Studies, Hannam University, Daejeon, 34430 South Korea

**Keywords:** Social support, Susceptibility, Preventive behavior, Pandemics

## Abstract

**Background:**

The COVID-19 pandemic hit Italy much harder than South Korea. As a way of explaining the different impact in the two countries, this study examines the moderating role of social support on the relationship between perceived susceptibility and preventive behaviors in the two countries.

**Methods:**

Surveys were conducted in South Korea (*n* = 1396) and Italy (*n* = 487) of participants aged 50 to 89 years.

**Results:**

South Koreans felt higher levels of perceived social support than their Italian counterparts. As would be expected, greater perceived susceptibility was associated with increased preventive behavior. Furthermore, a significant three-way interaction effect was found for perceived susceptibility, social support, and country. For Italians, a person who feels him/herself highly susceptible will increase preventive behaviors, if there is a lot of social support. On the other hand, for South Koreans, those with a low level of susceptibility perform more preventive measures than people with a high level of susceptibility if there is a lot of social support.

**Conclusions:**

This study provides insights into how cognitive factors, such as susceptibility and severity, as well as social and environmental factors can be taken into account, and the public be told the real risk and given behavioral guidelines when a pandemic is approaching. Given the critical role of social support as a coping mechanism in crisis situations, societies should mull over ways to increase emotional and instrumental support.

## Background

Preventive behaviors are critical to managing epidemic infectious disease; yet these behaviors have been far from universal in response to COVID-19. Factors that affect preventive behavior were widely studied [1–8], also with regard to mental health [9–11], and perceived susceptibility [12]. For example, beliefs about the consequences of preventive behaviors such as social distancing and face mask wearing were significant predictors of engaging in such activities [3]. However, the effects of social and cultural environments on individuals’ susceptibility to COVID-19 have seldom been investigated.

In fact, social support could play a significant role in affecting individual perceptions of susceptibility. One important feature of the Covid-19 pandemic has been its impact on the social environment. Public policies seeking to limit the spread of COVID-19 including social distancing and quarantines have increased stress from social isolation. This has highlighted the importance of social support [1, 9] which relates to help in caregiving and situational coping. However, how different circumstances embody the adoption of preventive behaviors against COVID-19 remains to be assessed.

In this paper, we compare adoption of preventive behaviors in two countries whose COVID-19 experiences have been very different—Italy and South Korea. Both are similarly sized; Italy has 61 million and South K33orea 51 million inhabitants [13]. However, as of June 7, 2021, Italy has had 4,232,428 confirmed cases and 126,523 deaths, while South Korea has had 144,637 cases with 1974 deaths (Johns Hopkins University & Medicine, 2021) [14]. How prevalence and prevention are linked remains to be assessed.

Preventive behaviors are key to reducing the spread and impact of COVID-19. Since the World Health Organization (WHO) declared a pandemic on March 11, 2020, there are still high numbers of confirmed cases and deaths with new variants such as Omicron and BA 5. To alert the public to keep practicing preventive measures, it is important to note that people respond to health threats according to their conception of how susceptible they are [15, 16] and how severe the damages are [17, 18]. Susceptibility and severity are two key factors of preventive behaviors, according to the Health Beliefs Model [17, 18] and the Theory of Planned Behavior [19].

Comparing Italy and South Korea provides an opportunity to examine the role of susceptibility in countries with widely differing severity as well as allowing study of susceptibility in relation to the social and cultural environment. Italy demonstrates high COVID-19 severity, in contrast to South Korea with its small number of cases and deaths. As to susceptibility, individual’s perceived susceptibility is a key determinant of engagement in preventive behaviors. For instance, US residents who underestimated the risk of contracting COVID-19 showed lower preventive behaviors [20].

Another important feature of the COVID-19 pandemic has been the importance of social support for coping [21]. Studies on the effects of social support suggest that it could play a role in responding to a health threat [22–24]. Social support could have differing effects, and this study seeks to examine varying effects of social support on individual COVID-19 judgements. The paradoxical thing was that government-imposed lockdowns, quarantines, limited gatherings, and closed public places hindered the efficacy of social support when it was needed most [25].

In the present study, a formative measure incorporating four aspects of social support taken from the perspective of the prospective recipient was developed tapping emotional support, relational support, private support, and instrumental support [22, 26–28]. Social support from significant others is associated with better health [23], with enhancing psychological well-being in both Western and Asian societies [29], with improving physical activity and quality of life in Korea [22, 30, 31]. Thus, in the present research, a positive main effect of social support on adoption of preventive behaviors for COVID-19 is expected.

### Comparison of Italy and South Korea

We chose to study Italy and South Korea—two countries with cultures exhibiting strong social bonds. In Italy, attachment to the family is a key value, more constant than any other cultural value [32]. Family gatherings are frequent and provide both emotional and economic support) [33]. The value of familism is the cherished jewel of Italian identity. For critics, however, familism resembles egotism, in that it is only extended to one’s next of kin. This can often prevent identification with larger entities in society, such as one’s community, region, class, or nation. This can prevent or inhibit organized social action on these levels [32].

Family solidarity and relationships are also essential part of the culture in South Korea, although family culture in the country has changed in response to rapid modernization [34]. Social support from family and friends is manifest in the long-term commitment to the in-group, fostering strong relationships and loyalty [35]. In Korea, however, high levels of collectivism, social bonds, and belongingness beyond the family play key cultural roles [36–38].

Individuals from different cultures show different levels of willingness to seek social support, as well as different perceived benefits from social support from those close to them [34, 39]. Individuals in Asian cultures are generally less willing to seek explicit social support for coping with their stress than those in European cultures [40], and they are less aided by social support [39].

Given the importance of social ties in Italy and South Korea, social support should impact individual response to the COVID-19 pandemic. Previous studies have found that social support has acted as a coping mechanism in crisis situations as well as a factor in resilience following disaster [41, 42]. For example, higher levels of social support are correlated with improved health behaviors [43] and sleep quality under quarantine during the COVID-19 pandemic [44]. Moreover, social support is negatively associated with individuals’ negative emotions, such as anxiety, depression, and loneliness during COVID-19 [11, 41, 43, 44]. While expectations for providing social support are more broadly based in South Korea, people in that society are less willing to seek such support; so no expectation is advanced regarding the relative impact of social support on preventive behavior in the two countries.

### Risk perception regarding COVID-19

Severity [45, 46] and susceptibility affect perceived risk [47], and they can be criteria for decisions on preventive measures [15, 16, 48]. Generally, according to the Health Belief Model (HBM), perceived susceptibility and perceived severity are the primary factors that affect proactive health behavior. Demographic and psychosocial factors such as age, social support, personality, self-efficacy, knowledge, and education are also predictive of health behaviors [17, 49].

The appraisal of severity is a collective judgement [50], but the appraisal of susceptibility compares the ego with all relevant others, so many people will consider themselves not to conform to the general trend. The perception of severity leads to assessments of humankind and its inclination to yield to a particular virus or any other disease agent. The perception of susceptibility involves the same assessment, as well as a number of self-assessments that are likely to differ from the assessments that others have formed of the subject [50]. These self-assessments tend to be both idiosyncratic and better protected in the subject’s mind and are therefore not as open to social influence as other factors. Moreover, the consideration of susceptibility can be subject to the optimistic bias effect [51].

People who think that they may easily be infected and fear the consequences will show more preventive behaviors than those who think they are safe or that suffering would be tolerable. This should be tested in relation to the COVID-19 outbreak. More importantly, given the stark differences in confirmed cases and mortality rates between Italy and South Korea, we expect different levels of perceived social support and for it to play different roles in the two societies. The devastation of Italy and its extremely high death toll leads us to expect high levels of perceived severity as a collective judgment. However, individual assessment of the likelihood of getting infected oneself varies.

Because severity is much higher in Italy, it may be more salient and have a greater impact in that country. We investigate the pattern of susceptibility and its impact on preventive behaviors in Italy versus South Korea. Of particular interest is the question of whether perceived social support moderates the effect of individual susceptibility on adoption of preventive behaviors toward COVID-19.

## Methods

We conducted cross-sectional surveys in South Korea (*n* = 1396) and Italy (*n* = 487) during the late fall 2020. The sampling frame in both countries was adults aged 50 years or older. As risk perception is closely associated with demographic factors, especially age [52, 53], we decided to focus on the older population (50 and older) who are objectively at greater risk for serious infection from COVID-19. In this way, differences in the patterns and logistics of social support [22] can be better controlled as well. In South Korea, participants were contacted in November 2020 by a leading survey firm that has access to a representative, country-wide panel using random sampling. In Italy, participants were recruited through snowball sampling from November to December 2020. The ethics committee at the Ewha Womans University in Seoul confirmed the study was outside the committee’s jurisdiction. The participants were 50 to 89 years old in Korea (M = 61.33, SD = 9.42) and from 50 to 83 years old in Italy (M = 59.87, SD = 7.08). In both countries, female respondents outnumbered males (Korea: 52.7%; Italy: 86.4%).

### Key measurements

Social support*,* as noted earlier has multiple definitions leading to varied operationalizations [26]. Overall, social support can be classified into emotional and instrumental support. Emotional support describes the impact a person has on his/her relatives and friends by speaking with them and listening to them. This relates to advice seeking, and it occurs in relation to health and in many social environments [22, 54]. Instrumental support focuses on the tangible, particularly physical assistance such as the assistance for those who are bed-ridden and thus may need help eating, performing hygiene, and receiving medication, and services such as transportation [27]. Finally, support can also tap close social ties. An individual would usually not prefer to have his/her health status discussed before the public [55–57]. Health is inherently a private matter, and inclination to keep health matters private limits the available social influence and social support to close ties such as one’s family [58].

In this study we used the existing six-item social support scale [27] in the context of COVID-19 crisis (Cronbach α = .92, M = 3.63, SD = .94). The scale used the format “How often is each of the following kinds of support available to you if you need it?” with a 5-point response scale (1 = none of the time, 5 = all of the time). Six items were: “Someone to help you if you were confined to bed;” “Someone to take you to the doctor if you needed it;” “Someone to share your most private worries and fears with;” “Someone to turn to for suggestions about how to deal with a personal problem;” “Someone to do something enjoyable with;” and “Someone to love and make you feel wanted.”

Preventive behaviors with regard to COVID-19 were measured using 16 items rated on a 5-point Likert scale (1 = strongly disagree, 5 = strongly agree), modified from extant studies [58, 59] were: “Avoided travel novel coronavirus infected areas,” “Washed hands with soap, hand sterilizer, and water,” “Used disinfectants,” “Avoided touching your eyes, nose, and mouth with unwashed hands,” “Avoided eating outside of the home,” “Stayed home when you were sick,” “Covered your cough or sneeze with a tissue, then throw the tissue in the trash,” “Avoided close contact with people who are sick,” “Wore a face mask,” “Avoided public transport,” “Avoided social events,” “Avoided going out in general,” “Avoided going to hospital or other healthcare settings,” “Avoided crowded places,” “Avoided contact with people who have a fever or respiratory symptoms,” and “Intend to comply with the government’s recommended actions” (Cronbach’s = .87, M = 4.23, SD = .51).

Perceived susceptibility was measured with four items rated on the same 5-point Likert scale as preventive behaviors. The items were: “Then novel coronavirus will spread widely in South Korea/Italy,” “I am more likely to get the novel coronavirus than other people,” “I believe I can protect myself against the novel coronavirus,” and “I believe I can protect myself against the novel coronavirus better than other people” (Cronbach’s α = .51, M = 3.21, SD = .54; the last two scales were reversed before entered into the models). The perceived susceptibility scale was modified from that used in previous studies [60–62].

Perceived severity was measured using two items rated on the same 5-point Likert scale as preventive behaviors. The items were: “My health will be severely damaged if I contract the novel coronavirus,” and “I think the novel coronavirus is more severe than the flu” (Cronbach’s α = .61, M = 4.36, SD = .70). The perceived severity scale was modified from those used in previous studies [60–62].

### Analysis plan

We used a hierarchical regression approach. The first block included demographic and health factors. The second block added severity and susceptibility. Then, the third block included social support, followed by the country factor in the fourth block. The fifth and sixth block included two way interaction terms and a three way interaction term, respectively.

That is, age, gender, education level, economic status, health condition, diagnosis of family members, and diagnosis of friends were used as covariates. Diagnosis of family members was checked with the question, “Has anyone in your household (excluding yourself) been diagnosed with COVID-19?” and diagnosis of friends was measured with the question, “Has anyone else you know been diagnosed with COVID-19?”

## Results

Table 1 presents the descriptive statistics for the control variables used in the analyses. The Koreans reported higher level of economic status and health condition than Italians: participants responded that their economic status was good, very good, or excellent (Italy: 38.6%, vs. South Korea: 72.4%), and their health condition as good, very good, or excellent (Italy: 59.1%, vs. South Korea: 84.8%). The percentage of diagnosis of family members (Italy: 9.9%, vs. South Korea: .3%) and diagnosis of friends (Italy: 73.1%, vs. South Korea: 7.9%) differ between Italians and South Koreans. Italians were nearly 10 times as likely to have friends or family who were diagnosed with coronavirus than South Koreans were.

**Table 1:** Descriptive statistics for control variables

Control Variables	South Korea		Italy	
	N	%	N	%
Age				
50–59	683	48.9	276	56.7
60–69	435	31.2	157	32.2
70–79	196	14.0	44	9.0
80–89	82	5.9	10	2.1
Gender				
Male	661	47.3	65	13.3
Female	735	52.7	421	86.4
Education Level				
No Formal Education	28	2.0	0	0
Primary School	135	9.7	3	.6
Secondary School/High School	294	21.0	241	49.5
Higher Diploma/Associate Degree	156	11.2	36	7.4
Bachelor	627	44.9	145	29.8
Master’s or Above	156	11.2	62	12.7
Economic Status				
Poor	41	2.9	83	17.0
Fair	344	24.6	216	44.4
Good	797	57.1	151	31.0
Very Good	200	14.3	35	7.2
Excellent	14	1.0	2	.4
Health Condition				
Poor	15	1.1	46	9.4
Fair	197	14.1	153	31.4
Good	740	53.0	212	43.5
Very Good	428	30.7	63	12.9
Excellent	16	1.1	13	2.7
Diagnosis of Family Members				
Yes	4	.3	48	9.9
No	1392	99.7	439	90.1
Diagnosis of Friends				
Yes	110	7.9	356	73.1
No	1286	92.1	131	26.9
Total	1396	100	487	100

Table 2 shows bivariate correlations among key variables. As predicted, positive relationships were found between perceived susceptibility and preventive behaviors (*r* = .13, *p* < .001) and between perceived severity and preventive behaviors (*r* = .34, *p* < .001). Those with high levels of perceived susceptibility and severity regarding COVID-19 were more likely to engage in preventive behaviors. Social support was positively associated with preventive behaviors (*r* = .06, *p* < .05).

**Table 2:** Bivariate correlations among key variables

	1	2	3	4
1. Susceptibility	1			
2. Severity	.16***	1		
3. Social Support	−.16***	.12	1	
4. Preventive Behaviors	.13***	.34***	.06*	1
Mean	3.21	4.36	3.63	4.23
SD	.54	.70	.94	.51

*p < .05, ***p < .001

Overall, Italians showed higher levels of perceived susceptibility (Italy: M = 3.44, SD = .50, vs. South Korea: M = 3.12, SD = .53), *t* (1881) = − 11.65, *p* < .001). Koreans showed higher levels of perceived severity than Italians (Italy: M = 4.18, SD = .85, vs. South Korea: M = 4.42, SD = .63), *t* (1881) = 6.60, *p* < .001). It is noteworthy that compared to the similar range of standard deviations for susceptibility (SD = .50 vs. .53, for Italy and South Korea), the variation in severity appeared larger for Italy, indicating a wider range of scores (SD = .85 vs. .63, for Italy and South Korea).

### Perceived social support during the COVID-19 pandemic: Italy vs. South Korea

We examined whether Italians and Koreans differed in terms of perceived social support. Overall, perceived social support was significantly different between Italians and South Koreans (Italy: M = 3.26, SD = 1.12 vs. South Korea: M = 3.76, SD = .83, *t* (1881) = 10.33, *p* < .001, effect size d = 0.54). South Koreans felt significantly higher levels of perceived social support than their Italian counterparts. This held true in all six items: “Someone to help you if you were confined to bed” (Italy: M = 3.09, SD = 1.30, vs. South Korea: M = 3.70, SD = .99), *t* (1881) = 10.78, *p* < .001, effect size d = 0.57); “Someone to take you to the doctor if you needed it” (Italy: M = 3.28, SD = 1.38, vs. South Korea: M = 3.82, SD = .97), *t* (1881) = 9.41, *p* < .001, effect size d = 0.50); “Someone to share your most private worries and fears with” (Italy: M = 3.31, SD = 1.39, vs. South Korea: M = 3.64, SD = .94), *t* (1881) = 5.85, *p* < .001, effect size d = 0.31); “Someone to turn to for suggestions about how to deal with a personal problem” (Italy: M = 3.34, SD = 1.35, vs. South Korea: M = 3.55, SD = .94), *t* (1881) = 3.78, *p* < .001, effect size d = 0.20); “Someone to do something enjoyable with” (Italy: M = 3.28, SD = 1.23, vs. South Korea: M = 3.91, SD = .92), *t* (1881) = 11.91, *p* < .001, effect size d = 0.63) and “Someone to love and make you feel wanted” (Italy: M = 3.28, SD = 1.52, vs. South Korea: M = 3.93, SD = 1.01), *t* (1881) = 10.56, *p* < .001, effect size d = 0.56).

### Social support on preventive behaviors in relation to susceptibility and country

To examine the moderating role of social support, hierarchical regressions were run. In the first block, age, gender, education level, economic status, health condition, diagnosis of family, and diagnosis of friends were included, which explained about 17% of the total variance (*R*^2^ = .174, df = 7, F = 56.27, *p* < .001). When the second block included severity and susceptibility, R^2^ increased from .174 to .280 (*R*^2^ = .280, df = 9, F = 80.81, *p* < .001). The third block added social support, which accounted for 28.8% of the total variance (*R*^2^ = .288, df = 10, F = 75.82, *p* < .001). The addition of country in block 4 increased R^2^ from .280 to .350 (*R*^2^ = .350, df = 11, F = 91.75, *p* < .001). Two-way interaction terms (susceptibility and social support, susceptibility and country, and country and social support) accounted for 35.4% of the total variance (*R*^2^ = .354, df = 14, F = 73.11, *p* < .001). The last block included the three-way interaction terms of susceptibility, social support, and country, which together explained 35.6% of the total variance (*R*^2^ = .356, df = 15, F = 68.68, *p* < .001).

The regression model showed no multicollinearity with the variance inflation factor (VIF), ranging from 1.11 to 2.38, and no correlation problem was seen between the residuals (Durbin-Watson = 1.799). As the final hierarchical regressions show in Table 3, severity (B = .27, *p* < .001), susceptibility (B = −.04, *p* < .05), social support (B = .08, *p* < .001), country (B = .41, *p* < .001), a two-way interaction term between susceptibility and country (B = .13, *p* < .01), and a three-way interaction term among susceptibility, social support, and country statistically significantly (B = .08, *p* < .05) predicted preventive behaviors.

**Table 3:** Effects of social support and susceptibility on preventive behaviors

Predictors		Preventive Behaviors					
		Model 1 B (t)	Model 2 B (t)	Model 3 B (t)	Model 4 B (t)	Model 5 B (t)	Model 6 B (t)
1	Age	.01 (6.17)***	.01 (5.34)***	.01 (5.64)***	.01 (5.23)***	.01 (5.23)***	.01 (5.23)***
	Gender	.25 (10.82)***	.22 (10.44)***	.23 (10.71)***	.17 (8.10)***	.17 (8.05)***	.17 (8.01)***
	Educational Level	.02 (1.55)	.02 (1.66)	.01 (1.59)	.01 (1.16)	.01 (1.11)	.01 (1.04)
	Economic Status	−.03 (− 2.02)*	−.05 (−3.72)***	−.07 (−4.55)***	−.04 (− 2.55)*	−.04 (− 2.49)*	−.04 (− 2.49)*
	Health Condition	−.01 (−.76)	.01 (1.00)	.01 (.34)	.03 (2.32)*	.03 (2.32)*	.03 (2.46)*
	Diagnosis of Family	−.10 (− 1.41)	−.08 (− 1.32)	−.07 (− 1.11)	.03 (.54)	.03 (.56)	.04 (.59)
	Diagnosis of Friends	−.31 (−11.42)***	−.33 (− 12.87)***	−.33 (− 13.05)***	−.09 (− 3.04)**	−.08 (− 2.76)**	−.08 (− 2.77)**
2	Severity		.24 (16.61)***	.23 (15.53)***	.27 (18.61)***	.27 (18.48)***	.27 (18.49)***
	Susceptibility		−.01 (−.49)	.00 (.16)	−.02 (−1.29)	−.05 (−2.22)*	−.04 (− 2.04)*
3	Social Support			.05 (4.76)***	.07 (6.27)***	.08 (5.86)***	.08 (5.75)***
4	Country				.43 (13.38)***	.41 (12.42)***	.41 (12.39)***
5	Susceptibility x Social Support					−.01 (−.26)	−.04 (−1.51)
	Susceptibility x Country					.11 (2.47)*	.13 (2.87)**
	Country x Social Support					−.03 (−1.55)	−.04 (− 1.96)
6	Susceptibility x Country x Social Support						.08 (2.16)*
	R^2^	.174***	.280***	.288***	.350***	.354***	.356***

Note: Gender (1 = male, 2 = Female); Diagnosis of family members/friend (1 = Yes, 2 = No); Country (0 = South Korea, Italy = 1)

*N* = 1883

B values are unstandardized regression coefficients, *p < .05. **p < .01. ***p < .001

Among covariates, age (B = .01, *p* < .001), gender (B = .17, *p* < .001), economic status (B = −.04, *p* < .05), health condition (B = .03, *p* < .05), and diagnosis of friends (B = −.08, *p* < .01) were significantly associated with preventive behaviors. That is, females, older people, those of low economic status, those in better health condition, and those with friends with COVID diagnoses were more likely to engage in preventive behaviors. More importantly, the results showed a significant three-way interaction effect (perceived susceptibility x social support x country: B = .08, t = 2.16, *p* < .05). That is, the interaction between susceptibility and social support was different in the two countries.

**Fig. 1:**
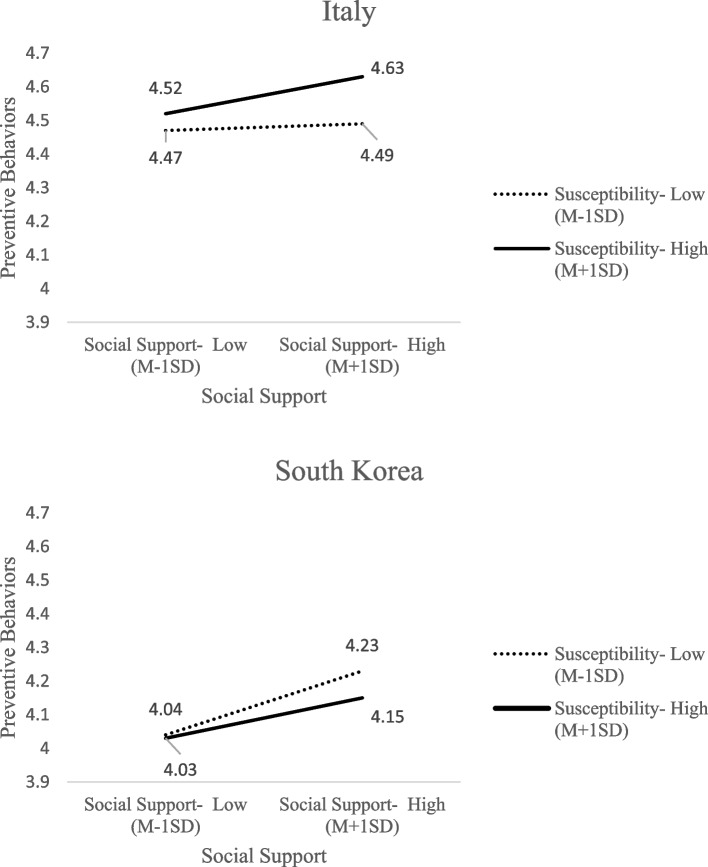
Three way interaction effect among susceptibility, social support, and country

Figure 1 shows the two patterns in Italy and South Korea. In Italy, the positive effect of social support was stronger for those with high susceptibility, who also demonstrated a significantly increased association between preventive behaviors and higher levels of social support (4.52 vs. 4.63; t = 3.14, *p* < .01). However, the association did not hold for persons with low susceptibility; for them, high social support did not go along with preventive behaviors: 4.47 vs. 4.49 (t = .35, *p* > .05).

By contrast, in South Korea, the positive effect of social support was stronger for those with low susceptibility. When individual’s perceived social support was low, no significant difference was seen in preventive behaviors between those with higher susceptibility (4.03) and those with lower susceptibility (4.04) (t = −.33, *p* > .05). The beneficial association of social support appeared stronger for those with low susceptibility in South Korea, increasing from 4.04 to 4.23 (B = .10, t = 5.51, *p* < .001).

## Discussion

This study compares Italy and South Korea and demonstrates that perceived risk is a key factor in predicting individuals’ preventive behavioral decisions. Social support was at different levels and produced different effects in the two countries. In Italy, a country with individualistic culture, the positive effect of social support was stronger for those with high susceptibility, while the boosting effect of social support was greater for those with low susceptibility in South Korea, a country with collectivistic culture.

First, women, older people, those with low economic status, those in better health condition, and those with friends with a COVID-19 diagnosis were more likely to engage in preventive behaviors. This result is somewhat consistent with previous studies of COVID-19 and H1N1 preventive behaviors. Females and older people were likely to have high risk perception and this factor was associated with preventive behaviors [62, 63].

Second, we found perceived susceptibility and severity of COVID-19 were positively associated with preventive behaviors, which is in line with previous studies [64, 65]. In the COVID-19 situation, those expecting to be easily infected and those fearing to suffer serious damage were more likely to engage in preventive behaviors. Despite the unprecedented and universal impact of COVID-19 across borders, the results point to the importance of individuals’ beliefs. When a health threat approaches, we ask ourselves how likely we are to catch the disease and how much we might suffer from it.

South Koreans reported higher levels of perceived social support than their Italian counterparts. Italy was the epicenter of the COVID-19 outbreak in Europe, recording extremely high mortality. COVID-19 was called Italy’s largest crisis since World War II [66]. Stark differences were noted between the Italian and South Korean responses to the crisis and outcomes [67]. Although both countries have a national healthcare system including universal healthcare coverage, emotional and instrumental support during the pandemic were quite differently perceived.

For Italians, the lowest social support was observed for the item: “Someone to help you if you were confined to bed” (Italy: M = 3.09, SD = 1.30, vs. South Korea: M = 3.70, SD = .99). Similarly, the item “Someone to take you to the doctor if you needed it” also had quite low ratings compared to the Korean data (Italy: M = 3.28, SD = 1.38, vs. South Korea: M = 3.82, SD = .97). The items for emotional support were slightly higher, such as “Someone to turn to for suggestions about how to deal with a personal problem” (Italy: M = 3.34, SD = 1.35, vs. South Korea: M = 3.55, SD = .94) and “Someone to share your most private worries and fears with” (Italy: M = 3.31, SD = 1.39, vs. South Korea: M = 3.64, SD = .94). Our numbers correspond to the dramatic reports from the dramatic situation in Lombardy in Northern Italy.

The different levels of perceived social support between Italy and South Korea highlights the importance of social support in a crisis. Individuals can cope and reduce the perceived severity of upsetting events when they have social support, boosting their protective mental health [28, 68]. Moreover, social support is negatively associated with anxiety and plays a role in protecting negative emotions [28]. Thus, social support from significant others, friends and family can improve individuals’ preventive behaviors toward COVID-19 (coping skills), as well as a supporting good emotional regulation [23, 28, 69].

However, we find that in the crisis that took place in Italy, availability of social support was limited which, in turn, reduces crisis management and deters timely response. Although individuals desperately need social support to cope with the crisis, limited access to social support only aggravates the difficult situation. Those results are in line with previous studies that found that social support is positively associated with health behaviors (e.g., [43]) and mental health (e.g., [28, 41, 43, 44]).

More importantly, this study found a significant three-way interaction effect among perceived susceptibility, social support, and country. That is, the effect of social support in relation to susceptibility was different across the two countries. For Italians, a person who feels him/herself highly susceptible will increase preventive behaviors, if there is a lot of social support. However, if there is a low level of susceptibility, additional social support does nothing, as shown in Fig. 1.

On the other hand, for South Koreans, those with a low level of susceptibility perform more preventive measures than people with a high level of susceptibility if there is a lot of social support. With low levels of social support, there was little difference between those with high and low susceptibility. The effect of social support was stronger for those with low susceptibility. It is noteworthy that the boosting effect of social support was greater for those with low susceptibility in South Korea, while the beneficial effect of social support was stronger for those with high susceptibility in Italy.

That is, in the case of South Korea, social support appeared to help those with low susceptibility to take more preventive behaviors in response to COVID-19. A recent study revealed that those who were unsure of their risk for COVID-19 infection were not concerned about community spread, and they did not understand the disease enough to be fearful about its effect [65]. Those who underestimate their susceptibility are the ones who need additional help or a trigger to adopt on appropriate preventive behaviors. Due to the importance of motivating those who are less concerned about their susceptibility, the results in South Korea indicate the necessity of social support, which can take the form of both instrumental and emotional support in crises.

By contrast, in Italy, social support was more meaningful only for those with high susceptibility. Those with high susceptibility are those who are already equipped to perform preventive behaviors due to their pre-existing beliefs. Figure 1 implies possible health disparities during the crisis in Italy. Even with the additional help of social support, those with low susceptibility were not motivated to take up preventive behaviors.

These results are in line with previous studies showing a widening of health disparities in crisis situations [70–72]. For example, during the COVID-19 pandemic, health disparities occurred by race/ethnicity, gender, and socioeconomic status, resulting in differences in screening, occurrence, treatment, and mortality [70]. Moreover, existing health disparities were widened by regional inequalities in healthcare resources and disease incidence in the COVID-19 pandemic in China [71].

Italy and South Korea differ in cultural values (e.g., individualism vs. collectivism), which may cause the different role of social support between two countries. The different roles of social support between Italy and South Korea accord somewhat well with a recent study comparing Taiwan (collectivism) and US (individualism) [1]. A comparison of the effects of early government communication during the COVID-19 pandemic showed effects of perceived susceptibility on preventive behaviors, moderated by perceived empowerment. That is, Taiwanese had higher perceived government empowerment than Americans, and the perceived government empowerment increased preventive behaviors through intrapersonal empowerment. The results show that during a public health crisis, individuals depend on broader information, such as government communications to achieve empowerment. Thanks to relatively successful and effective government communication in Taiwan, Taiwanese who had high government empowerment showed more appropriate behavioral actions. However, no such link was found for Americans, who felt less empowered.

These results have theoretical and practical implications. First, extant research has largely focused on the role of cognitive factors (e.g., perceived susceptibility and perceived severity). The current study points to the effects of social and cultural environments in relation to susceptibility to health threats. Second, the results highlight the boosting effect of social support, suggesting that increasing perceived social support can promote the preventive behaviors of people who perceive less susceptibility, at least in South Korea. However, the psychological mechanism or cultural differences that can explain why the relationship between perceived susceptibility and social support was different between Italy and South Korea remain to be examined.

The limitations of this study should be mentioned. First, it was conducted on a self-report survey using cross-sectional data. Sampling procedures in Italy cannot claim to have been representative of the country’s populations. As the COVID-19 pandemic is an ongoing phenomenon with a continual growth of cases and deaths, this could affect the perceived susceptibility and severity of COVID-19 at each specific point in time. Second, social support was based on a self-constructed scale which has not been validated. Neither was any of the measures validated for intercultural comparison. Thirdly, although this study adopted and modified the perceived susceptibility and perceived severity scales from previous studies [60–62], their levels of reliability were a bit lower in this study. Thus, further examinations are needed to investigate these scales. Also, considering the negative impacts of some preventive measures such as social distancing, further studies are needed to clarify differential effects of preventive behaviors. Finally, further study is warranted to understand the psychological mechanism or cultural differences that can address why the relationship between perceived susceptibility and social support was different between Italy and South Korea.

## Conclusions

This study provides insights into how health communication practitioners can take account of cognitive factors, such as susceptibility and severity, as well as social and environmental factors when developing health messages and campaigns. Results also underscore the role of media and government to inform the public of the real risks with behavioral guidelines to promote preventive behaviors. Most of all, given the critical role of social support as a coping mechanism in crisis situations, societies should mull over ways to increase emotional and instrumental support.

## Data Availability

The datasets used and/or analysed during the current study are available from the corresponding author on reasonable request.
